# Incidentally Discovered Duodenal Gastrointestinal Stromal Tumour (GIST): Operative Treatment and Problems After Surgery—A Case Report and Literature Review

**DOI:** 10.1155/crgm/5493240

**Published:** 2025-02-14

**Authors:** Peter Lüthje, Ilona Nurmi-Lüthje

**Affiliations:** ^1^Retired, Helsinki, Finland; ^2^Department of Public Health, Helsinki University, Tukholmankatu 8 B, Helsinki 00290, Finland

## Abstract

**Background:** Gastrointestinal stromal tumours (GISTs) are mesenchymal tumours of the digestive tract that can involve any part of the tract. The tumours can be harmless or life-threatening.

**Materials and Methods:** A case report of a surgeon who fell in a Finnish sauna, and he immediately felt that some ribs were broken. Magnetic resonance imaging and ultrasound showed three fractured ribs and an intrasplenic haematoma. Contrast-enhanced computed tomography (CT) demonstrated a small intrasplenic anomaly but no haematoma. Incidentally, an incidentaloma in the left adrenal gland was diagnosed. Three months later, a control CT scan was performed. The radiological findings on the adrenal gland and laboratory examinations matched those of a benign adenoma. Incidentally, a small duodenal tumour was diagnosed. At the same time, anaemia (haemoglobin: 104 g/L) and iron deficiency (ferritin: 8 μg/L) were noticed. An esophagogastroduodenoscopy showed an intramural tumour localised after the bulb-descending junction. Because the tumour was submucosal, the pathological diagnosis failed. Three months later, a radical surgical resection of the tumour with a resection margin of 2 mm and primary closing of the duodenum was performed. Pathological examination showed a well-circumscribed submucosal mesenchymal tumour with spindle cells. A tumour-free margin was uncertain. Immunohistochemistry findings showed a GIST. Due to the uncertain margin, an esophagogastroduodenoscopy control was planned at 2 years postoperatively. The patient disagreed with the decision and ordered a private control CT 3 months after the operation. The new CT found no local recurrence or metastasis. The patient contacted the head surgeon of the clinic, who ordered a 1-year postoperative CT. The 1-year follow-up CT finding agreed with the previous findings.

**Conclusion:** The aftertreatment of a radical-operated GIST is extremely important if histologic examination of the tumour-free margin is uncertain. In that case, CT controls should be considered once a year for at least 3 years.

## 1. Introduction

Gastrointestinal stromal tumours (GISTs) are the most common mesenchymal tumours of the gastrointestinal tract [1]. These tumours can involve almost any segment of the gastrointestinal tract, from the distal oesophagus to the anus, although the stomach is the most common site (> 50%) [1]. About 30% of GISTs are detected in the jejunum or ileum, 5% in the duodenum, 5% in the rectum and < 1% in the oesophagus [1]. Approximately 70% of GIST patients are symptomatic [2]. The most common clinical presentations are gastrointestinal bleeding and abdominal discomfort, and the average age of patients diagnosed with stromal tumours is 60 years [2].

From a recovery perspective, GIST varies from a small, harmless tumour nodule to a metastasising and life-threatening sarcoma [1]. Prognostic parameters are tumour size (maximum tumour diameter in cm) and localisation, mitotic rate per 50 high power fields (HPFs) (corresponding to 5 mm^2^) [1], and perforation of the tumour before or during the operation. A tumour size of < 2 cm with < 5 mitoses/50 HPF has a good prognosis [3]. Radical resection of the GIST with a 1-2 cm clear margin is sufficient treatment [4].

We present a case of an unexpected GIST diagnosed on contrast-enhanced CT after a falling accident.

## 2. Case Report

In September X, a white native 80-year-old man slipped and fell in a Finnish sauna, and his left chest hit the doorstep. Because the patient was a surgeon and traumatologist, he immediately felt that some ribs were broken. There was no macroscopic haematuria. To obtain precise information on the injury, thoracic and lumbar magnetic resonance imaging (MRI) and an abdominal ultrasound were performed in a private clinic 3 days later. The radiologist noticed three fractured ribs on the left side (costa 10, 11 and 12). He also noticed a small intrasplenic haematoma but no other injuries.

Because of the intrasplenic haematoma, the patient was sent to the emergency department of the University Hospital for a contrast-enhanced computed tomography (CT) of the thorax and abdomen. The CT demonstrated a small intrasplenic anomaly but no haematoma. Incidentally, an incidentaloma (11 × 11 × 13 mm) in the left adrenal gland was diagnosed. In the emergency department, the haemoglobin value was 111 g/L. It was assumed that the reason for the low haemoglobin value was the large haematoma and fractures of the ribs due to the falling accident (Table 1).

Some laboratory tests were privately performed earlier, and iron deficiency (ferritin: 21 μg/L) was diagnosed in March X (Table 1). The patient then started to use oral iron medication 100 mg twice a week.

The patient's daily medication was losartan 50 mg, simvastatin 10 mg, acetylsalicylic acid 100 mg (aspirin) and daily use of vitamin D supplements, 1600 IU in winter and 800 IU in summer months.

Two weeks after the CT in September X, an abdominal Doppler ultrasound was performed in the hospital in question. No spleen injuries were noticed.

Because of the incidentaloma, a visit to an endocrinologist was ordered. The endocrinologist ordered a new CT scan with intravenous contrast in December X. All laboratory tests ordered by the endocrinologists were normal.

Meanwhile, a colonoscopy was performed in a private clinic in December X, as the patient occasionally had diarrhoea. The findings were normal.

**Figure 1:**
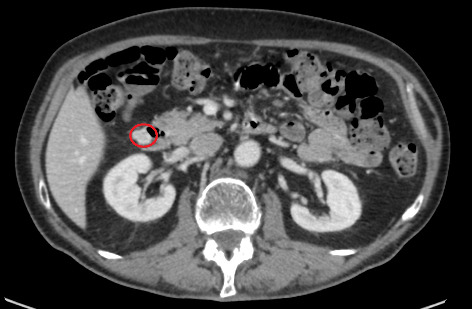
Axial abdominal intravenous contrast-enhanced computed tomography of the duodenal tumour (red circle). Helsinki University Hospital, Diagnostic Centre, Radiology, Helsinki, Finland.

In the new CT scan, the size of the tumour in the left adrenal gland was the same as in the first examination (11 × 11 × 13 mm). The diagnosis was a benign adenoma. Incidentally, in the new CT, a duodenal tumour (16 × 13 × 14 mm) was diagnosed (Figure 1). The tumour was already visible 3 months earlier in the first CT scan, but the radiologist did not notice this lesion. The radiologic diagnosis of the tumour situated in the lateral wall of the second part of the duodenum was a neuroendocrine tumour (NET).

According to the new laboratory examinations in December X, the haemoglobin was 104 g/L, and the ferritin level was only 8 μg/L (Table 1). The patient stopped the use of aspirin, assuming that it was the reason for the increasing anaemia. Two weeks later, the haemoglobin was 109 g/L (Table 1). All tumour markers were normal.

In January *X* + 1, an esophagogastroduodenoscopy was performed. The intramural duodenal tumour was localised after the bulb-descending junction. Because the tumour was submucosal, the pathological diagnosis failed. The endoscopist believed that the tumour was a NET or a GIST. A new endoscopic biopsy was planned.

The patient consulted a gastrosurgeon of the hospital who did not recommend a new biopsy but suggested an open laparotomy and a complete surgical resection. Because of anaemia (Hb 109 g/L) and iron deficiency (ferritin: 8 μg/L), an intravenous iron preparation (ferric carboxymaltose: 1000 mg) was given in January *X* + 1.

The case was discussed in a gastrosurgery meeting and a local resection with primary closing was recommended. In March *X* + 1, the patient underwent an open wedge resection of the duodenal tumour with simple closure of duodenal wall by two gastrosurgeons. The surgeons stated that the surgical resection of the tumour was complete with a clear margin. The resection margin was 2 mm.

The tumour size was about 1.5 cm. No intraperitoneal lymph node metastasis or liver metastasis was observed. The surgeon also removed one abnormally coloured diverticula of the colon transversum. The patient received cefuroxime 1500 mg intravenously once before and three times after the operation. The nasogastric tube was removed on the third postoperative day and the abdominal drain 1 day later. Parenteral nutrition stopped after 3 days. Oral intake started the same day.

The patient recovered uneventfully and was discharged from hospital after 5 days. One day later, however, an incipient wound infection was diagnosed. The patient started oral cefuroxime (500 mg × 3) daily. Because the local infection worsened, he contacted the emergency department of the hospital. However, the resident physician on duty was not worried about the infection. Thirteen days after the operation, the wound partially ruptured, and a lot of suppuration exuded from the wound. The bacterial culture showed a *Bacteroides fragilis* infection, and the oral antibiotic metronidazole (300 mg × 3) was prescribed for 10 days. Local use of silver content in dressing and lavage of the wound healed the infection in 3 weeks.

**Figure 2:**
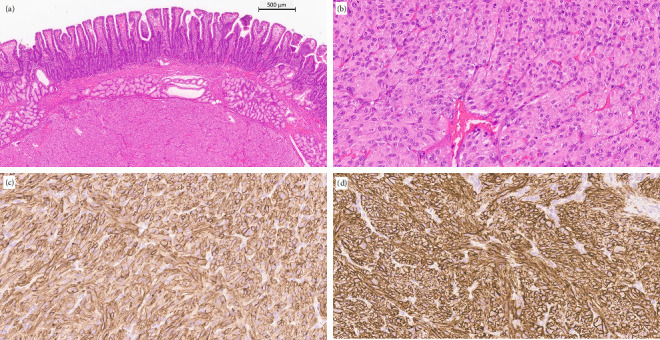
(a) Duodenal mucosa is intact. A mesenchymal tumour with minimal atypia is seen in the submucosa. Haematoxylin and eosin. (b) In the tumour, also epithelioid morphology was seen. Haematoxylin and eosin. (c) In immunohistochemistry, CD117 was positive and (d) DOG1 was positive. Helsinki University Hospital, Diagnostic Centre, Pathology, Helsinki, Finland.

Postoperative pathological examination showed a well-circumscribed submucosal mesenchymal tumour with spindle cell variants (Figure 2). The tumour was enucleated. A tumour-free margin was uncertain.

The immunohistochemistry findings of the tumour were as follows: DOG 1+, CD117+, CD34+, S-100-, SMA-, CKPAN- and desmin-. The tumour was a GIST.

The tumour size was 1 cm with < 1 mitoses/5 mm^2^, and the risk classification was group 1 (GIST 1) according to the Armed Forces Institute of Pathology criteria [5].

According to the pathological examination, there was no tumour in the diverticula. Some stool was found in the diverticula. The histological findings were also evaluated by a senior pathologist. The result did not change.

In April *X* + 1, the patient was informed by phone by a resident physician whom he did not meet in person that a 2-year postoperative esophagogastroduodenoscopy control was planned.

According to an experienced gastroradiologist, a waiting time of about 3 months after an operation for a new CT would be adequate because the postoperative operation area would be healed. Thus, a contrast-enhanced CT scan of the thorax and abdomen was privately performed in June *X* + 1. The new CT did not reveal any local recurrence or metastasis.

After a discussion with the main gastrosurgeon a 1-year postoperative control CT in March *X* + 2 was planned. The main gastrosurgeon also stated that an esophagogastroduodenoscopy control at 2 years postoperatively was the wrong decision.

Because the reason for the anaemia was unclear, the abdominal CT scans from September and December *X* were checked once again, but no anatomical reason for possible bleeding was observed. According to the specialist, the GIST was not the reason for the anaemia because the inner wall of the duodenum was intact.

In March *X* + 2, 1 year after the operation, new laboratory examinations were privately performed (Table 1). All tests were normal, including tumour markers serum carbohydrate associated antigen (19-9) and carcinoembryonic antigen. In the same month, a new abdominal contrast CT in the hospital was performed, which showed no changes compared with the previous CT examination. The patient received the CT scan results from the resident physician by phone, who also reported that no more CT controls were necessary. The patient did not agree with this decision because the resection margin of the tumour was uncertain. The resident discussed the problem with his senior. The conclusion was that the next CT scan in the hospital would be carried out at 2 years postoperatively. The patient was informed that the results of the next CT scan will be sent by digital outpatient service only.

## 3. Discussion

GISTs are quite rare. The annual incidence of GISTs has been estimated at 4–20 per million individuals in most countries [6–8]. Annual incidences vary in different countries because the diagnostic criteria have improved, leading to variations in diagnosis and recording [9]. Since 2021, according to the latest World Health Organization (WHO) classification [10], all GISTs should be classified as such [9], regardless of size, site of origin or mitotic index. Due to the present classification, annual incidences in different countries have increased.

In Finland in 2021, the annual incidence was 27 per million individuals (population: 5,548,241) [11]. There was a total of 152 cases (82 females, mean age 68.9 years and 70 males, mean age 68.2 years). The median ages were 69.7 years and 70.4 years, respectively [11]. Since 2021, all GISTs have been classified in Finland according to the new WHO classification [10].

Our duodenal GIST case is extremely rare. According to a study from Israel, there were only 4% duodenal GISTs among 93 patients with GIST [12]. In 24% of these 93 cases, the patients were symptomless [12]. In our patient, a duodenal GIST was incidentally diagnosed with an abdominal CT scan after trauma.

Immunohistochemistry studies in the present case showed antibodies DOG1 and CD117, which are the most sensitive and specific antibodies used in GIST diagnosis [13].

One important laboratory finding in the present case was anaemia, diagnosed 3 weeks after the abdominal CT scan (Table 1). Because the haemoglobin was 104 g/L, the patient stopped using daily aspirin. Two weeks later, the haemoglobin value was 109 g/L. He used 100 mg aspirin daily for the prevention of colorectal cancer for over 20 years [14]. He also had certain laboratory tests regularly done for many years, and for example, in August *X* − 1, his haemoglobin value was 142 g/L, but in March *X*, haemoglobin was only 126 g/L and ferritin 21 μg/L (Table 1). At that time, the patient started to use 100 mg of oral iron therapy every second day. The reason for the anaemia could be the use of daily aspirin, which is associated with a 20% higher risk of anaemia among people over 65 years [15] and a decline in ferritin [16]. In addition, a recent study in the United States showed that the adjusted incidence of upper gastrointestinal bleeding increased 36% from 2016 to 2020 among patients aged ≥ 75 years who routinely used aspirin for primary cardiovascular prevention [17].

The other reason for anaemia could be the GIST. A recent study showed that, in half of the cases, bleeding was a common symptom of GIST, but only 35% of the cases had ulcerated mucosa [18]. The authors concluded that mucosal ulceration and gastrointestinal bleeding do not appear to be the sole reason for anaemia in GIST patients [18]. In our case, there was no mucosal ulceration. A similar case with anaemia and a submucosal GIST without mucosal ulceration was published previously [19].

We believe that the reason for anaemia in our patient was the GIST. The patient used oral iron therapy every second day for about 20 months, but the ferritin level did not increase. The ferritin level was only 8 μg/L in January *X* + 1. After the intravenous iron infusion (ferric carboxymaltose: 1000 mg) in January and the operation in March *X* + 1, the ferritin levels were 100 μg/L in September *X* + 1 and 89 μg/L in March *X* + 2, respectively (Table 1).

In the present case, the gastrosurgeon could completely remove the duodenal tumour with a 2 mm resection margin. However, in the postoperative pathological examination, the tumour-free margin was uncertain. This was the reason the patient requested postoperative CT controls, which should be considered yearly for 3 years [20]. The ESMO guidelines defines radical surgical resection of localised GISTs as an excision whose margins are clear of tumour cells at least at the site of origin in the gastrointestinal tract [9]. Several articles in the literature recommend a macroscopic resection margin of about 1-2 cm and at least 1 cm to achieve a radical surgical resection surgery [4, 21]. Tumour rupture during the operation is an important prognostic factor for the estimation of recurrence risk and malignant behaviour of GIST [22]. In case of tumour rupture, micrometastatic disease can be presumed to exist. This increases the risk of relapses [23]. According to Cananzi et al. [24], the prognosis is influenced by tumour rupture rather than by the status of surgical margins.

In case of primary resection with positive margins by histopathological assessment, a careful re-evaluation should be undertaken by an experienced pathologist to exclude false-positive results [21]. Decision about possible reoperation should be individualized, considering a balance between the risk of reoperation, the possible benefits and the aggressiveness of the primary tumour [21].

According to the latest recommendations for low-risk tumours, the benefit of routine follow-up is not known [9]. In selected cases, abdominal CT scans or MRIs should be carried out every 6–12 months for 5 years [9]. In this case, an abdominal CT scan is the best alternative because the primary diagnosis was done with a CT scan.

Thoracic CTs are not necessary because all residual tumours appear in the abdominal cavity [20]. If the CT shows GIST recurrence, a 3-year adjuvant imatinib treatment is necessary [25]. A multicentre study conducted in Finland, Germany, Norway and Sweden (*n* = 400) suggests that approximately 50% of deaths could be avoided during the first 10 years of follow-up after surgery with this treatment [25].

In our case, the presurgery and surgery clinical processes were good, but the resident physician-led telephone follow-up service was not satisfactory. In the case of potential cancer, an outpatient clinic follow-up visit, when the patient can meet the responsible surgeon in person, is the best practice. If the histological findings are uncertain, the best expert for further procedures is the surgeon in charge of the primary operation.

## 4. Conclusions

GISTs are mesenchymal tumours of the digestive tract. They can occur at any portion of the gastrointestinal tract. The tumours can be harmless or life-threatening. Radical surgical resection is the gold standard option for GISTs. In patients with a high risk of recurrence, despite macroscopically completed surgery, 3 years of adjuvant imatinib treatment 400 mg/day is necessary. Follow-up examination should be based on the decision of an expert meeting.

## Figures and Tables

**Table 1:** Laboratory test results before and during the treatment process (year *X* = injury year).

Laboratory test	Reference	08/*X* − 1	03/*X*	09/*X*	01/*X* + 1	01/*X* + 1	03/*X* + 1	09/*X* + 1	12/*X* + 1	03/*X* + 2
Haemoglobin (g/L)	134–167	142	126	111	104	109	112	138	134	137
Leucocyte (E9/L)	3.4–8.2	5.1	3.2	4.7	4.1	4.2	3.9	4.0	4.5	3.6
Thrombocyte (E9/L)	150–360	200	175	172	363	208	171	208	171	162
Erythrocyte (E12/L)	4.25–5.7	4.42	4.01	3.39	3.68	3.95	3.76	4.34	4.11	4.21
Haematocrit (%)	39–50	41	37	33	32	34	34	39	39	40
MCV (fl)	82–98	93	92	93	87	87	89	89	96	95
RDW (%)	< 14	13	13	13	13	15	18	13	13	13
MCH (pg)	27–33	32	31	33	28	28	30	32	33	33
MCHC (g/L)	320–355	347	341	354	326	317	334	357	340	343
Creatinine (μmol/L)	60–100	90	—	76	—	82	70	77	—	80
ALP (U/L)	35–105	49	53	—	—	58	42	50	50	72
Folate (mmol/L)	> 8	—	—	—	11.8	—	—	—	—	—
Ferritin (μg/L)	20–195	—	21	—	—	8	—	100	83	89
S-25OHD (nmol/L)	> 50	—	93	—	—	—	—	—	—	116
CRP (mg/L)	< 4	—	—	< 4	—	< 4	15	—	—	—
Calcium–ion (mmol/L)	1.16–1.3/L/ph 7.4	—	—	—	1.24	—	1.09	—	—	—
ALT (U/L)	< 50	17	12	—	—	11	13	25	27	27
Folate (nmol/L)	8.8–42.4	—	—	—	—	11.8	—	—	—	—
B12-TC2 (pmol/L)	145–570	—	—	—	146	—	—	—	—	—
Albumin (g/L)	34–45	—	—	—	—	38	30	—	—	—
Bilirubin (μmol/L)	0–20	—	—	—	—	8	11	—	—	—
Amylase (U/L)	0–65	—	—	—	—	51	51	—	—	—
INR	1.0	—	—	—	—	1.0	—	—	—	—
CA 19-9 (kU/L)	< 26	—	—	—	—	5	—	—	—	8
S-CEA (μg/L)	0–5	—	—	—	—	< 1	—	—	—	< 1
K (mmol/L)	3.3–4.9	—	—	—	—	4.1	3.8	—	—	4.1
Na (mmol/L)	137–145	—	—	—	—	139	141	—	—	141
PT (%)	70–130	—	—	—	—	—	82	—	—	—
Blood sugar level (mmol/L)	4–6	5.4	—	—	—	—	—	5.6	—	5.5
A1C test (mmol/mol)	20–40	32	—	—	—	—	—	32	—	31

*Note:* A1C test = blood sugar over the past 3 months, B12-TC2 = active B12 vitamin, K = serum potassium, Na = sodium concentration.

Abbreviations: ALP = alkaline phosphatase, ALT = alanine aminotransferase, CA 19-9 = cancer antigen 19-9, CEA = carcinoembryonic antigen, CRP = C-reactive protein, INR = international normalized ratio, MCH = mean corpuscular haemoglobin, MCHC = mean corpuscular haemoglobin concentration, MCV = mean corpuscular volume, PT = prothrombin time, RDW = red cell distribution width, S-25OHD = serum 25-hydroxyvitamin D.

## Data Availability

The data used to support the findings of this study are available from the corresponding author upon reasonable request.
